# The Pediatric Anesthesia Parent Satisfaction (PAPS) Questionnaire: A Focused Review of its Development, Validation, Cross-Cultural Adaptation, and Clinical Utility

**DOI:** 10.7759/cureus.113067

**Published:** 2026-07-21

**Authors:** Seni Potsangbam, Rupesh M Thete, Abdelaziz Yehya, Sanjot Ninave, Tuhin Mistry, Abhijit Nair

**Affiliations:** 1 Department of Anesthesiology, Jawaharlal Nehru Institute of Medical Sciences, Imphal, IND; 2 Department of Anesthesiology, Shri Rawatpura Sarkar Institute of Medical Sciences and Research, Nava Raipur, IND; 3 Department of Pediatric Surgery, Ibra Hospital, Ibra, OMN; 4 Department of Anesthesiology, Jawaharlal Nehru Medical College, Datta Meghe Institute of Higher Education and Research (Deemed University), Wardha, IND; 5 Department of Anesthesiology, Ganga Medical Centre and Hospitals Pvt Ltd, Coimbatore, IND; 6 Department of Anesthesiology, Ibra Hospital, Ibra, OMN

**Keywords:** parent satisfaction, pediatric anesthesia, pediatric-anesthesia, postoperative period, questionairre, survey research

## Abstract

Patient satisfaction has become a central metric in evaluating healthcare quality. However, its use in pediatric anesthesia is less explored due to unique challenges in reporting satisfaction by a pediatric surgical patient. As a result of this, parental satisfaction is utilized as a validated proxy. To fill the gap of not having a validated tool for parent satisfaction, the Pediatric Anesthesia Parent Satisfaction (PAPS) questionnaire was developed, based on the American Society of Anesthesiologists Committee on Practice Management recommended questions. The tool was developed following a rigorous, expert, and parent-informed development process. This focused review attempts to summarize the available literature on the PAPS questionnaire, covering its development, psychometric validation, cross-cultural adaptation, and clinical application. Four articles meeting the inclusion criteria were identified. The original 17-item instrument demonstrated acceptable internal consistency (Cronbach's alpha above 0.7) and a three-factor structure involving physical symptoms, communication, and professionalism, with a pronounced ceiling effect. Two translations achieved excellent psychometric performance (Cronbach's alpha 0.937 and 0.96, respectively), confirming the instrument's cross-cultural robustness. Clinical application revealed consistently high satisfaction in high-income settings (more than 95%), with lower satisfaction observed only in one study (77.7%). The reasons for higher satisfaction were non-anxious parental status, male sex, urban residence, employment, and sedative premedication. Across all settings, postoperative symptom management and parental preparation for induction and emergence emerged as consistent areas for improvement. We conclude that PAPS is a globally applicable, psychometrically effective tool with considerable scope for improvement for continuous quality improvement in pediatric anesthesia care.

## Introduction and background

Patient satisfaction has emerged as a cornerstone of modern healthcare quality assessment. Over several decades, the shift toward patient-centered care has fundamentally transformed how health systems evaluate their performance. In the present era, patient satisfaction is no longer a peripheral concern but a central metric that determines departmental and organizational reputation and adherence to evidence-based practices, and it is an important area of research [1-4].

Pediatric anesthesia presents an additional and distinctive layer of complexity. Perioperative environments, particularly anesthetic involvement, are unfamiliar and stressful for children and their families. Pediatric patients show a wide spectrum of distress behaviors in response to anesthesia, including separation anxiety, protest, and violent behavior. For obvious reasons, it is not possible to assess patient satisfaction in this cohort [5-7]. This limitation necessitates using parental responses as a proxy for the child's satisfaction, a practice now validated and well-established in the pediatric anesthesia literature [8,9]. Studies have demonstrated that children's perioperative experiences are closely linked to and substantially influenced by their parents' experiences, meaning that parental satisfaction serves not merely as a substitute measure but as a clinically meaningful outcome in its own right [10,11].

To address this gap, Milliken-Glabe and colleagues developed the Pediatric Anesthesia Parent Satisfaction (PAPS) questionnaire in 2016 at Children's Hospital Colorado in Denver, United States [12]. Since its publication, the PAPS has garnered immense interest as the first comprehensive questionnaire to investigate parent satisfaction regarding children undergoing various pediatric surgeries. This was followed by validated translations and cross-cultural adaptations in other languages, and studies published from diverse settings like Indonesia [13], Norway [14], and Ethiopia [15].

This focused review aims to synthesize the available literature on the PAPS questionnaire, which includes the development process, analysis of its psychometric properties, and cross-cultural validation in various languages, ethnicities, and healthcare settings.

## Review

Methodology

We chose a narrative review design over a systematic review or meta-analysis because the PAPS questionnaire is a relatively new tool with a limited but methodologically diverse body of literature. The goal of this review was not to quantify the effect sizes but to systematically follow the development, validation, and clinical utility of PAPS in various settings. We included studies that described the development, validation, cross-cultural adaptation, or clinical application of the PAPS questionnaire or its translated versions. We restricted our inclusion to articles available in the English language only. We excluded studies in which tools other than PAPS were used to evaluate parent satisfaction. We also excluded expert opinion articles, conference abstracts, and unpublished reports. We searched PubMed, Scopus, and Ovid databases for articles related to PAPS. Four articles fulfilled the inclusion criteria for this focused review [12-15]. A summary of all the included studies is provided in Table 1. A summary of the psychometric properties of the included studies is provided in Table 2.

**Table 1:** Summary of included studies PAPS: Pediatric Anesthesia Parent Satisfaction

Study/Year	Country	Aims of the study	Study design	Sample size (n)	Nature of surgery	PAPS version used	Number of items	Response scale	Timing of questionnaire administration
Milliken-Glabe et al., 2016 [12]	United States	Development, implementation, and initial validation of PAPS	Cross-sectional	250	Elective	Original version- developed in this study	17	5-point Likert scale	Immediate postoperative
Sari et al., 2023 [13]	Indonesia	Cross-cultural translation and validation of Indonesian PAPS	Cross-sectional	125	Elective and emergency	Indonesian translation of PAPS was validated in this study	17	5-point Likert scale	Immediate postoperative
Leonardsen et al., 2025 [14]	Norway	Multicentre clinical application of the Norwegian translation of PAPS	Multicentre, cross-sectional	234	Elective	Norwegian translation of PAPS back-translated, and validated in this study	15 statements, open-ended	5-point Likert scale	Postoperative period when the child is fully awake
Admass et al., 2022 [15]	Ethiopia	To evaluate the level of parental satisfaction and associated factors	Cross-sectional	238	Elective and emergency	Original English version	15 rating items	5-point Likert scale	Immediate postoperative

**Table 2:** Summary of various psychometric properties in included studies PAPS: Pediatric Anesthesia Parent Satisfaction, KMO: Keyser-Meyer-Olkin, CFA: confirmatory factor analysis

Study/Year	Response rate	Factor structure	Kaiser-Meyer-Olkin/Bartlett’s test	Reliability (Cronbach’s alpha)	Item-level validity	Discriminant validity	Ceiling effect
Milliken-Glabe et al., 2016 [12]	100%	Three factors: Physical symptoms, communication, professionalism	Not performed	All 3 composites > 0.70 (Cronbach's α & Raykov's ρ); range 0.72–0.82	Item-to-own > item- to-to-another composite correlation ; all p<0.05	Significant difference in physical symptoms & communication between OR vs procedure Centre (p < 0.05)	Pronounced: median 5, mean ≥ 4.8 for all items ; 0% in the bottom two boxes
Sari et al., 2023 [13]	Not stated	Two factors (CFA) explaining 64.99% total variance; factor loadings > 0.40	KMO = 0.937; Bartlett's χ² = 1283.452, p < 0.001 (very high adequacy)	α = 0.937 (Excellent internal consistency )	Pearson r: 0.531–0.796; all p < 0.001; all 'strongly valid.' (r > 0.35)	Not assessed	Not described
Leonardsen et al., 2025 [14]	Not calculated	Principal component analysis conducted; full factor structure reported separately	KMO = 0.94; Bartlett's p ≤ 0.001 (very high adequacy)	α = 0.96 (very high internal consistency )	Spearman r: 0.2–0.9; all p < 0.01–0.05	Pain and PONV demonstrated inter-hospital variation	Not prominent; disagree responses present for several items
Admass et al., 2022 [15]	100%	PAPS applied directly as they used the original English version	NA	Not performed	Not performed	Not assessed	Not observed at same magnitude, Overall satisfaction-77%

Development of the PAPS questionnaire

Background and Rationale for Instrument Development

Patient satisfaction following pediatric anesthesia remains challenging, as children are not capable of answering a questionnaire as adults. Before the introduction of the PAPS, the assessment of satisfaction with anesthesia services in pediatric populations lacked a standardized, psychometrically validated instrument [16-20]. There were, however, several tools available for assessment of anesthesia satisfaction in adult patients, like the Iowa Satisfaction with Anesthesia Scale [21] and the Leiden Perioperative Patient Satisfaction Questionnaire [22].

In a review article by Barnett et al. [1], the authors summarized the six available tools to assess satisfaction with pediatric anesthesia assessed from parents [23-28]. They concluded that the Pediatric Perianesthesia Questionnaire by Schiff et al. was the most robustly developed tool [28]. Although it was lengthy and complex, the high response rate in its development study indicates that it is acceptable to parents. The authors felt that the tool needs further evaluation in multiple centres to increase its acceptability.

The Original PAPS Instrument

The PAPS survey tool was developed by Milliken-Glabe et al. to provide the first evidence of validity and reliability for the survey questions, incorporating American Society of Anesthesiologists (ASA) recommended question types to evaluate parent satisfaction with pediatric anesthesia services, identify strengths, and identify potential areas for improvement [12]. They followed DeVille's scale development methodology, which is a structured process widely used in health-related instrument construction [29].

The development of PAPS began with the framing of satisfaction questions recommended by the ASA Committee on Practice Management (COPM), developed through expert consensus but not exclusively adapted for pediatric populations, and was not independently validated either. These questions were then presented to a multidisciplinary group of anesthesiology and quality improvement experts, who reviewed them, modified the language and content to suit a pediatric surgery and anesthesia setting, and finalized the items deemed appropriate and clinically useful. Ten anesthesiologists subsequently reviewed the questionnaire and, based on their feedback and comments, made changes. Thereafter, a separate review was conducted involving 10 parents in the post-anesthesia care unit (PACU), who assessed and commented on the clarity and comprehensibility of questions. The questionnaire was revised based on the comments and suggestions, but keeping the number of items unchanged.

The resulting PAPS questionnaire comprised 17 items in total: one question regarding the location of the surgical procedure, 15 closed-ended rating items, and one open-ended question that allowed comments from the parents on the anesthesia experience (Table 3). The 15 rating items essentially covered the periods before anesthesia, after anesthesia, and during the surgery, and the conduct of the hospital team and anesthesia team. Each item was rated on a five-point Likert scale ranging from one (disagree very much) to five (agree very much), allowing for graded rather than binary responses.

**Table 3:** Pediatric Anesthesia Parent Satisfaction (PAPS) Survey. The survey has one question regarding the location of the surgical procedure, 15 closed-ended questions, and one open-ended question to be completed by the respondent. Source: Milliken-Glabe et al., 2016 [12]; reproduced with permission from the Publisher (John Wiley & Sons), and after the license was obtained through Copyright Clearance Center’s RightsLink® service. License number: 6298880159959, license date: June 30, 2026.

S. No.	Information requested.
1	The anesthesia team answered all my questions before my child's surgery.
2	I was satisfied with the amount of information provided by the anesthesia team.
3	The information given to me by the anesthesia providers was understandable.
4	I was satisfied with the way my child fell asleep and woke up from anesthesia.
5	The anesthesia team explained to me how my child might feel physically and emotionally after anesthesia and surgery.
6	I felt my child's pain was well controlled after surgery.
7	I felt my child's nausea and vomiting were well controlled after surgery.
8	My child's privacy was respected at all times by the anesthesia team.
9	The anesthesia staff we met for my child's surgery were professional and respectful.
10	The anesthesia team behaved professionally and respectfully toward my child.
11	The anesthesia team paid attention to my concerns regarding my child's care.
12	I was satisfied with the care my child received from the anesthesia team.
13	I know who the anesthesiologist (physician) was and his/her role in my child's care.
14	I would recommend this anesthesia team to others in my family.
15	My child received the highest quality care during this surgical experience.
	Please tell us more about your anesthesia experience.
	Room PACU, Procedure Center PACU)

The PAPS was administered to 250 English-speaking parents in the main operating room PACU and the procedure center PACU at the Children's Hospital Colorado, before discharge [12]. Parents of children under 18 years of age undergoing elective surgeries were included, after ascertaining that the parents were able to comprehend (read and understand) English. This study achieved a 100% response rate. Descriptive statistics revealed strongly skewed response distributions for all 15 least favorable response options for any item, thus producing a pronounced ceiling effect. Although this points towards a high satisfaction in this single-institution, English-speaking, elective day-surgery sample, it also strongly points towards a psychometric limitation, which was acknowledged by the authors, with the explanation being partly due to the homogeneous study population. Exploratory factor analysis with Promax rotation, applied to 12 of the 15 rating items after excluding the three overall satisfaction items, revealed three underlying measurement constructs, labeled as physical symptoms, communication, and professionalism.

Internal consistency reliability was assessed using both Cronbach's alpha and Raykov's rho, with all three composite scales achieving values above the conventional threshold of 0.7, supporting acceptable internal construct reliability. Item-to-own composite scale correlations exceeded item-to-other composite scale correlations across all items, providing further evidence of construct validity. Discriminant validity was demonstrated through statistically significant differences in two of the three composite scores between the main operating room and the procedure center.

The original PAPS was developed and validated in a single institution in the United States, involving an English-speaking sample drawn from a largely elective, day-surgery unit. While its psychometric properties were acceptable, the generalizability of any patient-reported outcome instrument to different linguistic, cultural, and healthcare contexts was not foolproof. Cross-cultural adaptation requires a systematic examination of whether the conceptual equivalence, semantic meaning, and measurement properties of an instrument are preserved when it is deployed in a population with different cultural norms, educational structures, and healthcare experiences, along with linguistic translation.

Indonesian Validation

The Indonesian validation of the PAPS was conducted at Sardjito General Hospital, Yogyakarta, Indonesia, by Sari et al. [13], using a three-stage translation protocol. Forward translation from English to Indonesian was performed utilizing the Indonesian Language and Culture Learning Services (INCULS) at the Faculty of Art and Humanities, Universitas Gadjah Mada, Yogyakarta. An anesthesiology expert then reviewed the translated version for clinical accuracy and readability, after which a backward translation into English was performed by INCULS to verify semantic and conceptual equivalence with the original. Pilot testing involved four anesthesia consultants, five patients' families, six nurses, and seven anesthesia residents, whose suggestions were utilized to finalize the Indonesian version of the PAPS, which then underwent formal validity and reliability testing subsequently.

The Indonesian version redefined the term 'parent,' which in the original English version referred to the biological parent. The Indonesian team adopted a broader definition that extended to caregivers, guardians, and any other person who accompanied the patient through the perioperative period. This definitional expansion reflected both Indonesian family structures and the operational realities of pediatric surgical care in the local setting, where a biological parent is not always the accompanying adult. A total of 125 subjects were enrolled with a mean age of 6.78 ±5.05 years, of which 56% were male. The majority of procedures were elective (96.8%), and ASA classification II was the most common (56.8%).

Validity and reliability findings: Item-level validity was evaluated using Pearson's correlation coefficient. With correlation coefficients ranging from r = 0.531 to r = 0.796 and all significant at p < 0.001, all 15 items in the Indonesian version showed strong validity. The suitability of the dataset for factor analysis was confirmed by the Kaiser-Meyer-Olkin (KMO) measure of sampling adequacy, which was 0.937 and categorized as very high, along with a significant Bartlett's test of sphericity (χ² = 1283.452, p < 0.001). Two factors accounted for 64.985% of the variance, according to confirmatory factor analysis, and all factor loadings were higher than the 0.40 threshold needed for a good construct. The 15 items' communality values ranged from 0.449 to 0.797, indicating low to moderate shared variance among items. This finding is consistent with the instrument's multidimensional but coherent structure.

Cronbach's alpha was used to evaluate internal consistency reliability; the result was a coefficient of 0.937, which is considered excellent by standard interpretive benchmarks. This result indicated that the Indonesian version maintained comparable measurement precision despite variations in language, culture, and patient population. It also closely matched the original PAPS validation, where Cronbach's alpha and Raykov's rho both exceeded 0.7 across all three composite scales. The authors came to the conclusion that the Indonesian version of the PAPS was appropriate for regular clinical and research use in Indonesia since it was valid and reliable for evaluating parental satisfaction with pediatric anesthesia services [13].

Norwegian Validation

The Norwegian adaptation of the PAPS was conducted by Leonardsen et al. [14] at three hospitals of varying sizes in Southeastern Norway, after the translation and cultural adaptation. The English version of the PAPS was back-translated into Norwegian, after which face and content validity were confirmed with a pilot study involving 10 parents. Psychometric testing was conducted using data collected from Hospital 3 (n = 112) during an initial phase of the study. The principal component analysis yielded a KMO value of 0.94 and a statistically significant Bartlett's test (p ≤ 0.001), thus confirming an adequate sample size. Spearman's correlation analysis was suggestive of a significant association between items (p < 0.01 and p < 0.05), with coefficients ranging from 0.2 to 0.9. The resulting Norwegian PAPS (NPAPS) was thus considered valid. The Norwegian version preserved the original 15-item closed-ended structure and five-point Likert response format, as in the original English version. Demographic questions specific to the Norwegian context were added, including the parents' prior experience of accompanying a child to surgery and their relationship to the patient. In Norway, the item referring to the "anesthesiologist" in the original English version was adapted to refer to both the anesthesiologist and the nurse anesthetist, reflecting the Norwegian perioperative staffing model in which nurse anesthetists and anesthesiologists share primary responsibility for anesthetic care.

No formal sample size calculation was done. The inclusion criteria comprised parents of children < 18 years of age undergoing surgery under general anesthesia. Surgeries included ear-nose-throat, gastric, urologic, and eye. Parents of children undergoing emergency surgeries and parents who did not understand Norwegian sufficiently were excluded. Data were collected from all three hospitals, and a total of 234 parents responded. The median age of pediatric patients was seven years (IQR 4-11), and the most common surgical specialty was ear-nose-throat (37.8%), followed by urologic (21.9%) and gastrointestinal (11.2%) procedures.

The Norwegian validation demonstrated very high internal consistency, with a Cronbach's alpha of 0.96. The overall psychometric profile of the Norwegian PAPS, combining strong factor analytic results, high item intercorrelations, and excellent internal consistency, supported its suitability for both clinical quality monitoring and research purposes in the Norwegian perioperative setting.

Ethiopian Study

Admass and colleagues conducted an institution-based cross-sectional study at a comprehensive specialized referral hospital in Gondar, Ethiopia, enrolling 238 parents of children undergoing elective and emergency surgery between March and June 2021, achieving a 100% response rate [15]. The PAPS questionnaire was administered alongside the modified Yale Preoperative Anxiety Scale [30] and the State-Trait Anxiety Inventory [31], enabling concurrent measurement of child and parental anxiety. Admass et al. reported an overall parental satisfaction of 77.7% (95% CI: 72.3%-82.4%), substantially lower than rates reported in high-income settings but representing a meaningful improvement over the 59.8% previously reported from another Ethiopian institution [32].

Item-level analysis revealed that the highest satisfaction was reported by parents in higher-income settings. The highest ratings were for professional conduct, privacy respect, and overall care quality. However, variables like postoperative symptom management (40.8%) and parental preparation during induction and emergence (32.8%) were reported as the lowest. Using a multivariable logistic regression, the authors identified that non-anxious parental status (adjusted OR (AOR) 3.45), male sex (AOR 2.58), urban residence (AOR 2.90), employment (AOR 4.09), and receipt of sedative premedication by the child (AOR 2.30) were the highest predictors of parent satisfaction. Admass et al. suggested that to enhance parent satisfaction and also to reduce anxiety of pediatric patients perioperatively, there is a need for targeted preoperative support for rural, female, and anxious parents, and the use of premedication as a routine strategy [15].

Clinical findings: application of the PAPS across settings for assessing satisfaction

In the study by Milliken-Glabe et al., more than 95% of parents reported being satisfied or very satisfied with pediatric anesthesia services [12]. The study was conducted in a university hospital in the United States, which was a well-resourced, tertiary academic center with a selected elective day-surgery population. The Norwegian multicenter study reported similarly high satisfaction, with 96.4-97.4% of respondents across all three hospitals agreeing that their child had received the highest-quality care during the surgical experience, and also agreeing on the professional conduct, information quality, and respect for privacy [13]. In the Ethiopian study by Admass et al., the parental satisfaction with anesthesia services was 77.7% (95% CI: 72.3-82.4%) [15], which is significantly lower than the previous two. Admass et al. mentioned that these discrepancies could be due to underdeveloped anesthesia services in low-income countries, a shortage of skilled personnel, and inadequate infrastructure. Only the Ethiopian study evaluated the factors affecting satisfaction levels using multivariate logistic regression [15]. The reasons were less anxious parents, male parents, urban resident parents, employed parents, and parents whose kids received sedative premedication. The summary of satisfaction results in the included studies is presented in Table 4.

**Table 4:** Summary of satisfaction results in included studies CI: confidence interval; OR: odds ratio; AOR: adjusted odds ratio; PONV: postoperative nausea/vomiting

Study/Year	Overall satisfaction rate	Highest scoring domain	Lowest scoring domain	Predictors of parent satisfaction	Statistical methods employed for investigating the association
Milliken-Glabe et al., 2016 [12]	>95% satisfied	Professionalism	Pain control, PONV	None mentioned	Not examined
Sari et al., 2023 [13]	Not reported (as it was a translation and validation study))	All 15 items were strongly valid	Not applicable (as it was a translation and validation study)	Not mentioned	Not examined
Leonardsen et al., 2025 [14]	96.4-97.4%	Professionalism, privacy, and quality of care	Physical/emotional explanation	None identified	Linear regression; Kruskal-Wallis test for inter-hospital differences
Admass et al., 2022 [15]	77.7% (95% CI: 72.3–82.4%)	Privacy, overall care	Pain control, PONV	Non-anxious parent, male, urban stay, employed	Bivariable and multivariable logistic regression; OR and AOR with 95% CI

Strengths and limitations of PAPS

PAPS was not derived randomly or in an institution- or region-specific manner. It was developed from questions recommended by ASA COPM, which gives it a degree of face validity and professional legitimacy. This makes PAPS unique and different from other questionnaires related to pediatric anesthesia. PAPS was developed following the structured DeVilles scale development process, along with a comprehensive literature review, expert panel review, pilot testing, and refinements at every stage to improve and refine.

The reading level of the questionnaire was equivalent to a grade level of 4.8 using the Flesch-Kincaid Readability Index [33], 5.6 using the Automated Readability Index [34], and 9.7 using the SMOG Index [35,36]. This suggests that it can be easily understood even by low-educated respondents. The questionnaire is age-independent, thus reducing further bias, as the parents are the ones who will respond and not the patient. The PAPS can be used either in a printed format or an electronic format, making it more user-friendly and analysis-friendly. Both the Norwegian and Indonesian languages are linguistically and culturally different. In spite of that, the translation and validation of PAPS in these languages demonstrated cross-cultural validity and high internal consistency.

However, there are several limitations of PAPS as a tool. The PAPS is administered in the immediate postoperative period, typically in the PACU before discharge. This timing has practical advantages and is also easy for the researchers, especially after day-care surgeries, the clinical situation is fresh in the parents' memory, and the opportunity to capture responses is at its greatest before the family leaves the PACU; however, it also introduces a characteristic bias. On many occasions, parents could have waited for months for a particular surgery and were finally relieved once it was over. This could inadvertently inflate the ratings they give. On the other hand, if the child comes to PACU with pain, presents with agitation or nausea/vomiting, the responses could be lower even though these are common and manageable events in the PACU. The ASA COPM also recommends that satisfaction surveys should ideally be administered within two weeks of the procedure to allow for a more realistic picture.

PAPS does not record the child's firsthand experience with anesthesia and surgery, but it records parental experience. Although parental proxy response is a recognized and accepted consideration, considering young children's developmental limitations, it is crucial to acknowledge that the perioperative experiences of parents and children do not always coincide. In the original English version, the authors did not collect sociodemographic or clinical data from respondents, such as parental age, education, income, child diagnosis, or anesthesia duration, which is a potential limitation. Later in the Ethiopian and Norwegian study, the issues were addressed to some extent. A comprehensive psychometric evaluation of any evaluation tool requires evidence not only of internal consistency and construct validity but also of test-retest reliability. This is missing in the original English version and the subsequent validations.

## Conclusions

The PAPS questionnaire is an important tool that fills the gap that existed for the measurement of satisfaction with anesthesia services for pediatric surgical patients, which was developed through a rigorous process involving expert inputs from clinicians and feedback from parents, using ASA COPM-endorsed questions, and is suitable for quality improvement and research. The tool proved its global application as a result of its cross-cultural adaptations.

The PAPS tool has enough scope for further cross-cultural adaptation and language translation across the world. There is enough scope for refining and improving the tool, which can thus benefit the pediatric anesthesia community with a reasonably validated, globally applicable, and clinically sensitive tool that can be used for continuous improvement of pediatric anesthesia services to improve parent satisfaction and derive excellent patient outcomes.
